# Constitution and By-Laws of the American Society of Dental Surgeons

**Published:** 1844-09

**Authors:** 


### Constitution and By-Laws

### OF THE AMERICAN SOCIETY OF DENTAL SURGEONS.

### ARTICLE I.

The objects of this Society are to promote union and harmony among all respectable and well informed Dental Surgeons; to advance the science by free communication and interchange of sentiments, either written or verbal, between members of the Society, both in this and other countries; in fine, to give character and respectability to the profession, by establishing a line of distinction between the truly meritorious and skilful, and such as riot in the ill-gotten fruit of unblushing impudence and empiricism.

ARTICLE II.—*Of the Name of the Society.*

The Society shall be known and designated by the name and title of "The American Society of Dental Surgeons."

ARTICLE III.—*Of the Officers of the Society.*

Sec. 1. The Officers of the Society shall consist of a President, and three Vice-presidents, a Recording and a Corresponding Secretary, a Treasurer, a Librarian, an Executive and Examining, and a Publishing Committee.

Sec. 2. The election of the above named officers shall be by ballot, at a regular annual meeting of the Society—a majority of votes determining the election.

ARTICLE IV.—*Of Members of the Society.*

Sec. 1. There shall be two classes of Members—the first acting Members or Fellows, who shall assent to and adopt this Constitution, and pay into the treasury the annual dues required by it. The second shall be known and recognized as Honorary Members, or Fellows.

Sec. 2. Each and every applicant on being admitted to Membership, shall pay into the Treasury the sum of fifteen dollars, which shall entitle him to the Diploma of the Society, and also include the first years' subscription of five dollars. And he shall also pay five dollars at every annual meeting thereafter.

Sec. 3. Any Member who shall neglect to pay his annual dues to the Society, for three meetings of the same, shall be notified, and if still he does not settle, his name shall be stricken from the list of Members.

Sec. 4. Each and every Acting Member shall be required to attend the Society at least once in three years, unless excused by the Society.

ARTICLE V.—*Of Election to Membership.*

All applications for Membership shall be in writing, and recommended by at least three members of this Society, who are personally acquainted with the professional qualifications, and moral character of the applicant, and are willing to avouch for the same, or submit to an actual examination in all the departments of Dental Surgery, by this Society.

ARTICLE VI.—*Of the Expulsion of Members.*

Any Member of the Society may be expelled for immoral conduct, malpractice in business, or other sufficient cause, on motion of one member, seconded by another, at any regular meeting of the Society, in which case a majority of three-fourths of the members present, shall be required.

ARTICLE VII.—*Of the Meetings of the Society.*

The Meetings of the Society shall be held annually by adjournment, from time to time, and from place to place, agreeably to the will of the Society.

ARTICLE VIII.—*Of the Resources of the Society.*

The Society may receive contributions of money, books, or other property, which may be either used or sold in aid of its purposes.

ARTICLE IX.—*Of the Disposition of the Funds of the Society.*

The funds of the Society may be appropriated to the purchase of lands, tenements, chemical or philosophical apparatus,—the publishing of books, tracts, and other papers and documents; and to such charitable objects as shall hereinafter be enumerated.

ARTICLE X.—*Of the Distribution of the Effects of this Society, in the event of its final Dissolution.*

Sec. 1. In case a dissolution of the Society shall at any time be proposed, a meeting shall be called specially for that purpose; and the consent of three-fourths of its members be required to effect it.

Sec. 2. Should the Society thus be dissolved by its own act, the property belonging to it shall be sold by order of the President, or by any three Fellows who shall obtain authority from a majority of the living and subscribing members, and the assets of sale shall be equally divided among the subscribing members.

ARTICLE XI.—*Of the indispensable Qualifications of Candidates who have never yet entered upon professional practice.*

Sec. 1. The candidate shall be at least twenty-one years of age; shall have a good English education; shall produce evidence of unexceptionable moral character; and shall have studied and practiced for the full term of two years with some practical dentist known as such to this Society.

Sec. 2. No candidate for membership, who has the diploma of any Dental College, regularly chartered in any of the United States, shall be subjected to re-examination by the Examining Committee of this Society; but shall be entitled to a Diploma by complying with the By-Laws.

ARTICLE XII.—*Of the Quorum required to transact business; and of the contingency of there being no quorum present at any meeting.*

Sec. 1. Seven acting Members, or Fellows, of the Society, besides the President or Chairman, shall be necessary for the transaction of business, at the opening of any annual meeting.

Sec. 2. In the event that a quorum cannot be obtained at any regular meeting of the Society, the time of meeting on the following year shall be the second Tuesday of August, and the place the same as on the preceding year.

ARTICLE XIII.—*Of the Charitable Objects of the Society.*

Sec. 1. Any surplus monies in the treasury, may be appropriated, at any regular meeting of the Society, for the aid and relief of the widows and orphans of deceased members; or for the benefit of living members reduced to want, by sickness or other calamity; it being provided that no such appropriation shall be made without the consent of two-thirds of the members present.

SEC. 2. Other charitable or patriotic appropriations may be made at any regular meeting of the Society, by a majority of three-fourths of the members present.

SEC. 3. The President may, at any time, give pecuniary assistance to any member, or to the surviving family of any deceased member, out of any monies in the treasury, deposited there by benevolent individuals.

ARTICLE XIV.—*Of Alterations and Amendments of this Constitution.*

Any alteration, amendment, or revision of this Constitution, may be made at any regular meeting of the Society, by the joint assent of three-fourths of the Fellows present at such meeting.

### BY-LAWS.

### ARTICLE I.—*Of the Duties of Officers.*

SEC. 1. It shall be the duty of the President to preside at all meetings of the Society, when present; to sign the diplomas conferred on members during his term of office; to confer all honorary distinctions awarded by the Society, whether degrees of Dental Surgery, medals or other testimonials; and to draw upon the Treasurer for all monies appropriated by the Society.

SEC. 2. It shall be the duty of the first Vice-president to preside at all meetings of the Society in the absence of the President; and to perform the other prescribed duties of the President, in case of his death, resignation, sickness, or other disability.

SEC. 3. It shall be the duty of the second and third Vice-presidents to perform the duties of the above named officers, during their absence or inability.

SEC. 4. A President *pro tem.* shall be appointed to preside at any meeting, when the President and Vice-presidents are all absent.

SEC. 5. It shall be the duty of the Recording Secretary to note, in a book kept for the purpose, all the proceedings of the Society, during the term of his office, and to furnish copies of any parts thereof, at the order of the President or his substitutes; and also, to deliver the said book and proceedings to his successor in office. He shall also keep the seal of the Society, together with the copperplate, from which diplomas, certificates, or degrees, are struck; also all the records belonging to the Society, designed to be preserved; and he shall furthermore countersign all diplomas, and set the seal of the Society to all instruments requiring the same; he shall also notify every member of the time and place of meeting of the Society, at least two months before the time of said meeting.

Sec. 6. It shall be the duty of the Corresponding Secretary to conduct the domestic and foreign correspondence of the Society, in accordance with the advice of the President, or the express will of the Society, communicated to him by the Recording Secretary, and at his own discretion, in cases of emergency, provided it does not interfere with any of the prescribed rules of the Society.

Sec. 7. It shall be the duty of the Treasurer to keep all the monies of the Society committed to his trust; to pay them over to the order of the President; and to keep a correct account of the same in a book or books kept for that purpose.

Sec. 8. It shall be the duty of the Librarian to keep the books belonging to the Society's Library; to obtain insurance on the same; and to take receipts of all persons who receive books from his hands by order of the Society; and in case of failure of the member receiving such book, to return it in twelve months, he shall forfeit, and pay to the Society, four times its price.

Sec. 9. It shall be the duty of the Executive and Examining Committee to examine the accounts of the Treasurer, and report the same to the Society at its annual meetings; to see that all the rules and regulations of the Society are duly enforced and executed; and it shall also be the duty of such committee to inquire into the professional reputation and character of all candidates proposed, or who may apply for membership, and report the same to the Society, which being satisfactory, such candidates shall be ballotted for, for admission into the same.

Sec. 10. It shall be the duty of the Publishing Committee to superintend the printing and publishing of all books, tracts, and other documents issued by the Society.

ARTICLE II.—*Of the Emoluments of Office.*

No officer shall receive any emoluments unless in cases of special appropriation, excepting the Recording and Corresponding Secretaries, whose duties it shall be to attend the Meeting of the Society next subsequent to their election; and who shall be entitled to their travelling expenses and the sum of five dollars per diem during their attendance at the sessions of the Society.

ARTICLE III.—*Of the Powers vested in the Vice-presidents.*

Each of the Vice-presidents may, if there be a sufficient number of the members of this society residing in his immediate vicinity, convene them at any time, and when so convened, they shall have power to elect such officers from among their own number as may be necessary for the transaction of whatever business of a local nature may come before them; and it shall be obligatory on them to report their proceedings regularly at each annual meeting of the parent society.

ARTICLE IV.—*Of the Privileges of Members.*

Sec. 1. Each and every acting Member, or Fellow of the Society, shall be entitled to debate and vote on all questions agitatedat any meeting thereof, agreeably to the By-laws and Standing Rules and Regulations of the Society.

SEC. 2. Each and every Honorary Member shall be entitled to a Certificate of Membership, to a seat at all meetings of the Sociey ; shall have the privilege of debating all questions not involving pecuniary expenditure ; shall not be eligible to any office, but have all other privileges of an active member.

### ARTICLE V.—*Of the Appointment of Members to certain duties.*

SEC. 1. It shall be the business of the society, at each annual meeting, to appoint six of its Members to prepare Dissertations on some subjects connected with the profession ; and also one Member to deliver an opening Address at the subsequent meeting.

SEC. 2. It shall moreover be the duty of the Society, from time to time, to appoint certain individuals to prepare Essays or any other Documents ordered by the Society.

### ARTICLE VI.—*Of Awarding Premiums.*

SEC. 1. This Society may award premiums to members, for dissertations on subjects that may at any time be specified by the Society at an annual meeting; also, for important improvements in Mechanical Dentistry, and the manufacture of Incorruptible Teeth; and also for any other suitable object.

SEC. 2. The question of preference in all such cases, shall be determined by the judgment of a Special Committee, appointed by the Society.

SEC. 3. Unsuccessful dissertations shall be at the disposal of their respective authors.

### ARTICLE VII.

SEC. 1. No member shall speak more than twice to any question, without express permission of the Society, and then only fifteen minutes at each time.

SEC. 2. Any member shall be liable to be called to order for personal recrimination of any other member; or for wandering in his remarks from the subject of debate.

### ARTICLE VIII.

These By-Laws may be altered or amended, at any annual meeting of this Society, by a vote of two-thirds of the members present.

